# Predictive and Prognostic Molecular Factors in Diffuse Large B-Cell Lymphomas

**DOI:** 10.3390/cells10030675

**Published:** 2021-03-18

**Authors:** Stefano A. Pileri, Claudio Tripodo, Federica Melle, Giovanna Motta, Valentina Tabanelli, Stefano Fiori, Maria Carmela Vegliante, Saveria Mazzara, Sabino Ciavarella, Enrico Derenzini

**Affiliations:** 1 Division of Haematopathology, European Institute of Oncology, IEO IRCCS, Via Ripamonti 435, 20141 Milan, Italy; federica.melle@ieo.it (F.M.); giovanna.motta@ieo.it (G.M.); valentina.tabanelli@ieo.it (V.T.); stefano.fiori@ieo.it (S.F.); saveria.mazzara@ieo.it (S.M.); 2 Tumor Immunology Unit, University of Palermo, 90133 Palermo, Italy; claudio.tripodo@unipa.it; 3 Tumor and Microenvironment Histopathology Unit, IFOM, the FIRC Institute of Molecular Oncology, 20139 Milan, Italy; 4 Hematology and Cell Therapy Unit, IRCCS-Istituto Tumori ‘Giovanni Paolo II’, Viale Flacco 65, 70124 Bari, Italy; mc.vegliante@oncologico.bari.it (M.C.V.); s.ciavarella@oncologico.bari.it (S.C.); 5 Division of Haemato-Oncology, European Institute of Oncology, IEO IRCCS, Via Ripamonti 435, 20141 Milan, Italy; enrico.derenzini@ieo.it; 6 Department of Health Sciences, University of Milan, Via di Rudinì 8, 20146 Milan, Italy

**Keywords:** diffuse large B-cell lymphoma, gene expression profiling, next-generation sequencing, classification, diagnosis, prognosis, therapy

## Abstract

Diffuse large B-cell lymphoma (DLBCL) is the commonest form of lymphoid malignancy, with a prevalence of about 40% worldwide. Its classification encompasses a common form, also termed as “not otherwise specified” (NOS), and a series of variants, which are rare and at least in part related to viral agents. Over the last two decades, DLBCL-NOS, which accounts for more than 80% of the neoplasms included in the DLBCL chapter, has been the object of an increasing number of molecular studies which have led to the identification of prognostic/predictive factors that are increasingly entering daily practice. In this review, the main achievements obtained by gene expression profiling (with respect to both neoplastic cells and the microenvironment) and next-generation sequencing will be discussed and compared. Only the amalgamation of molecular attributes will lead to the achievement of the long-term goal of using tailored therapies and possibly chemotherapy-free protocols capable of curing most (if not all) patients with minimal or no toxic effects.

## 1. Classification

Diffuse large B-cell lymphoma (DLBCL) is the most common form of lymphoid malignancy, with a prevalence of about 40% worldwide [1]. It consists of medium or large B-lymphoid cells in which the nuclei are the same size as or larger than those of normal macrophages, or more than twice the size of those of normal lymphocytes, with a diffuse growth pattern [1]. The concept of DLBCL has undergone fine-tuning over time, as is clear from the comparisons between the REAL and WHO classifications (third, fourth, and revised fourth editions) [1,2,3,4]. This produces some apparent terminological discrepancies throughout the text, which reflect the time of publication of each reference.

In the Revised Fourth Edition of the WHO Classification of Tumours of Haematopoietic and Lymphoid Tissues, DLBCL is subdivided into morphologic variants, molecular subtypes, and distinct disease entities (Table 1) [1]. Nevertheless, about 70% of all DLBCLs lack features allowing their inclusion into one of the diagnostic categories listed in Table 1 [1]. These cases are collectively termed as “not otherwise specified” (DLBCL-NOS) [1] and are conventionally treated with the chemoimmunotherapy regimen R-CHOP [5,6].

Based on a recent survey of 3550 DLBCL patients who mostly underwent R-CHOP with curative intent, the 5-year overall survival and cumulative incidence of relapsed/refractory disease corresponds to 65.3% and 23.1% of cases, respectively [6]. Thus, there is still an unmet need for optimal therapy for a significant proportion of DLBCL-NOS patients.

In recent years, DLBCL-NOS has been the object of the extensive application of high-throughput technologies, which has led to the identification of prognostic/predictive factors that are increasingly entering daily practice.

Although DLBCL-NOS is the main focus of this review, the borders between DLBCL-NOS and high-grade B-cell lymphoma (HGBCL) (Table 1) will also be discussed. In fact, it is not uncommon to encounter cases that could be regarded as DLBCL-NOS but are ultimately classified as HGBCL due to the detection of double or triple hits (D/TH) of *MYC*, *BCL2*, and/or *BCL6* (HGBCL-D/TH) by FISH, as underlined by Sehn and Salles in their review on DLBCL published in the *New England Journal of Medicine* on 4 March 2021 [7] (see below).

## 2. Gene Expression Profiling

### 2.1. Cell of Origin (COO)

At the beginning of this century, using gene expression profiling (GEP) Alizadeh and coworkers first reported that DLBCLs could be divided into two main subtypes with a gene signature related to the germinal center B-cell (GCB) and activated B-lymphocytes from the peripheral blood (ABC), respectively [8]. Such a distinction, not feasible on morphological grounds, had an important prognostic impact. In fact, the GCB forms had a significantly more favorable response to chemotherapy (CHOP) than those of ABC. This corresponded to a clear-cut difference in terms of overall and progression-free survival (OS and PFS, respectively). This subdivision was subsequently confirmed using cohorts consisting of hundreds of cases, and maintained its value in the era of chemoimmunotherapy [9,10,11]. By expanding the number of profiled cases, a third group between those of GCB and ABC emerged and was indicated as unclassified (U), corresponding to about 15% of DLBCLs [9,10,11]. Besides prognostic value, the distinction between GCB and ABC subtypes has biological relevance as it corresponds to different genetic aberrations as well as pathway perturbations (as detailed in the following).

The main limitation of conventional GEP was the need for fresh or frozen (FF) samples, which were available for a small minority of patients followed up at reference centers. Therefore, many attempts were made to find surrogates for GEP through the search for immunohistochemical markers [12,13,14,15,16,17,18]. Several algorithms were proposed, with that of Hans et al. having the widest applications as it was based on the simple determination of CD10, BCL6, and IRF4/MUM1 [12]. However, none of these algorithms met their goal, for several reasons: (a) a lack of correspondence with GEP data in the same patients; (b) variability in the preanalytical and immunohistochemical techniques (including antibody and antigen retrieval, detection systems, and automatic platforms); and (c) subjectivity in result interpretation [19,20].

In 2014, a new approach was proposed based on targeted digital GEPFF and was successfully applied to mRNA extracted from formalin-fixed, paraffin-embedded (FFPE) tissue samples (Lymph2Cx) [21]. In particular, a 20-gene panel (including 15 top genes and 5 housekeeping genes for normalization) was designed, which in 67 cases provided the same COO classification as conventional GEP from FF. Furthermore, the OS and PFS curves were over-imposable, irrespective of the type of GEP used (targeted digital vs. conventional). These preliminary results, which had been obtained by using the NanoString platform, were subsequently confirmed by independent studies based on several hundred cases [22,23,24,25]. The advantages of this approach over immunohistochemical algorithms are: (1) reproducibility in different laboratories; (2) the assessment of the absolute value of mRNA expressed by each gene; and (3) a lack of confounding factors (such as the variability of immunohistochemical techniques and subjective result interpretation). Moreover, targeted GEP subdivides DLBCLs-NOS into GCB, ABC, and U, like conventional profiling of FF samples. In contrast, immunohistochemical algorithms differentiate DLBCLs-NOS into GCB and non-GCB, with the latter group containing cases that are molecularly classified as GCB [21,22,23,24,25]. Interestingly, identical results were obtained by targeted profiling on different platforms and with different panels of genes confirming the prognostic relevance of the COO determination [21,22,23,24,25].

The COO determination provided less significant prognostic information when applied to cases enrolled in some trials [26,27,28]. This may be for several different reasons, i.e., (1) the adoption of protocols that are more intense than those used in real situations; (2) the selection of patients fit enough to await the completion of all the tests required for trial enrolment; and (3) the influence of other factors that can affect behavior within each subgroup defined by the COO.

The main limitation of targeted GEP applied to routine biopsies is the need for platforms which are not available in all pathology laboratories, unlike immunohistochemistry. This problem, as well as the test costs and need for basic bioinformatic skills, can be overcome by a hub-and-spoke organization, which is also required for the application of the array of molecular techniques at the basis of precision medicine (see below).

### 2.2. Key Genes

FISH analyses have shown that B-cell lymphomas, regarded as DLBCLs-NOS based on morphology and phenotype, could carry double or triple rearrangements of *MYC*, *BCL2*, and/or *BCL6* [1]. These cases, which overall have a significantly worse prognosis and may require therapies that are more intense than standard R-CHOP, are nowadays included in the provisional category of high-grade B-cell lymphomas with double/triple hits (HGBL D/TH). Based on this observation, FISH should ideally be applied to all DLBCL-NOS cases. As FISH analyses are rather expensive, attempts have been made to find surrogates for FISH results through immunohistochemistry. This has led to the identification of a group of DLBCL-NOS cases which show double expression of MYC and BCL2 at the protein level (the so-called double expressors (DEs)) [29,30]. According to the Revised Fourth Edition of the WHO Classification of Tumours of Haematopoietic and Lymphoid Tissues, at least 50% and 40% of neoplastic cells should express BCL2 and MYC, respectively, in order to consider a DLBCL-NOS patient as a DE [1]. However, discrepancies exist as to the reproducibility of the cut-off value of MYC positivity, which has been moved to 70% by some groups [31]. Most importantly, as there is no actual correspondence between the results of immunohistochemistry and FISH [32], these cases with MYC and BCL2 double-expression but lacking D/TH remain within the bounds of DLBCL-NOS but more often belong to the ABC/non-GCB subtype and require further studies to definitively assess their prognostic and/or therapeutic relevance [1,32].

In 2018, investigators from two groups used GEP signatures to identify high-risk patients with DLBCL in FFPE series. Sha et al. [33] used a Burkitt lymphoma-like signature, whereas Ennishi et al. [34] used a signature derived from genes differentially expressed between *MYC/BCL2* DH and non-DH GCB-DLCBLs. With their respective signatures, these investigators were, as expected, able to identify most DH lymphomas, as well as many non-DH lymphomas which were actually found in about half of the identified patients and showed a poorer response to standard chemoimmunotherapy. These findings suggest that many of the patients harbored genetic or even epigenetic alterations that produced similar gene expression changes in the tumor cells, as recognized by the respective signatures. This does not come as a surprise. In fact, activation of an oncogene or oncogenic pathway can be produced by multiple mechanisms besides *MYC/BCL2* DH, for example *MYC* upregulation through translocation and gene amplification. Apart from structural alterations, MYC expression or activity can be enhanced through transcriptional and posttranscriptional events.

Derenzini et al. [25] used a targeted GEP panel combining the Lymph2Cx signature for COO classification, with additional targets including *MYC, BCL-2*, and *NFKBIA* (the latter encoding for the IkB-α protein, an endogenous inhibitor of NF-kB signaling [35]), in 186 FFPE cases originally diagnosed as DLBCL-NOS from two randomized trials (discovery cohorts NCT00355199 and NCT00499018) and in three independent validation cohorts. By integrating the COO, *MYC/BCL-2* DE status, and *NFKBIA* expression, a three-gene signature was designed combining *MYC*, *BCL-2*, and *NFKBIA* (MBN signature). The high-risk (MBN Sig-high) subgroup, characterized by higher expression levels of *MYC* and *BCL-2* and a lower expression of *NFKBIA*, could be used to identify a significant fraction of ABC DLBCLs and the vast majority of double-hit cases, allowing for further risk stratification within the GCB/U subset. These results were validated in three independent series including Sha’s cohort based on the REMoDL-B trial [33,36], a phase III randomized trial investigating the efficacy of the addition of bortezomib to standard first-line chemoimmunotherapy. In line with the biological activity of bortezomib, which increases the protein abundance of IkB-α, leading to inhibition of NF-kB signaling [37], an exploratory ad hoc analysis of the latter cohort showed that the addition of bortezomib in the MBN Sig-high subgroup provided a progression-free survival advantage compared with standard chemoimmunotherapy. These data suggest that a simple three-gene signature based on *MYC*, *BCL-2*, and *NFKBIA* can refine the prognostic stratification in DLBCL.

Finally, Mottok et al. [38] developed a robust and accurate molecular classification assay (Lymph3Cx) for the distinction of primary mediatinal B-cell lymphoma (PMBCL) from DLBCL subtypes based on gene expression measurements in formalin-fixed, paraffin-embedded tissue. A probabilistic model accounting for classification error comprising 58 gene features was trained on 68 cases of PMBCL and DLBCL. Model performance was subsequently evaluated in an independent validation cohort of 158 cases and showed a high agreement of the Lymph3Cx molecular classification with the clinicopathological diagnosis of an expert panel (frank misclassification rate, 3.8%). In the authors’ view, Lymph3Cx represents a molecular tool that is potentially helpful for the diagnosis of PMBCL in light of the use of ad hoc therapeutic approaches [1]. In fact, on central review, cases enrolled in trials as DLBCL-NOS are not infrequently reclassified as PMBCL and vice versa, as seen in the authors’ experience at a reference center for several trials of the Italian Lymphoma Foundation.

## 3. Tissue Microenvironment (TME)

By means of a gene profiling analysis of nearly 500 FF DLBCL samples, in 2008 Lenz et al. first demonstrated that the expression of peculiar gene sets, namely “Stromal-1” and “Stromal-2” signatures, respectively correlated with good- and poor-outcome subgroups of R-CHOP-treated patients independently of COO [39]. Although they were selectively enriched in genes encoding extracellular matrix proteins and histiocyte infiltration (Stromal-1) or reflecting angiogenesis (Stromal-2), these signatures resulted mechanistically uninformative, and their practical use was limited by the lack of standardized GEP assays for FFPE samples. A number of subsequent research attempts were aimed at identifying TME-related prognostic factors, but none provided biomarkers reproducible enough to be translated into daily clinical practice [40,41,42,43].

To overcome this limitation, in 2018 Ciavarella and co-workers [44] generated a 1028-gene matrix incorporating the signatures of 17 cytotypes and applied the computational method CIBERSORT to deconvolve Lenz’s GEP dataset. The work clarified the prognostic associations between patient outcome and quantitative proportions of tumor-infiltrating cell types. A panel of 45 genes related to myofibroblasts (MFs), dendritic cells, and CD4+ T-cells was selected and digitally validated by a NanoString-based approach on an independent cohort of 175 FFPE DLBCLs from two randomized trials. All tissue samples consisted of pretreatment biopsies of advanced-stage nodal DLBCLs treated by comparable R-CHOP/R-CHOP-like regimens. The expression of the 45 TME genes positively correlated with better outcomes and predicted the patient risk of overall and progression-free survival. In a multivariate Cox model, the TME panel retained high prognostic performance independently of COO, and integration of the two prognostic factors (COO + TME) improved survival prediction. Finally, a model to assign single DLBCL cases to a “COO–TME” risk category was built and successfully applied to an independent cohort of 40 “real-life” cases.

In a parallel work, Staiger et al. [45] proposed a lymphoma-associated macrophage interaction signature (LAMIS) interrogating features of the microenvironment, once again using a NanoString assay applicable to FFPE. The clinical impact of the signature was validated in a cohort of 466 patients enrolled in prospective clinical trials at the German High-Grade Non-Hodgkin Lymphoma Study Group (DSHNHL). Patients with high expression of the signature (LAMIS-high) had shorter event-free survival (EFS), progression-free survival (PFS), and overall survival (OS). Multivariate analyses revealed independence from International Prognostic Index (IPI) factors in EFS (HR 1.7, 95%CI 1.2–2.4, *p*-value = 0.001), PFS (HR 1.8, 95%CI 1.2–2.5, *p*-value = 0.001), and OS (HR 1.8, 95%CI 1.3–2.7, *p*-value = 0.001). Multivariate analyses adjusted for the IPI factors showed the signature was independent of COO, MYC rearrangements, and double-expressor (DE) status. LAMIS-high and simultaneous DE status characterized a patient subgroup with dismal prognosis and greater probability of early relapse.

Beyond the prognostic value of TME, only a few studies have provided comprehensive biological insights into the putative link between B-cell genomics and functional patterns of immune and stromal components while suggesting new rationales for future therapeutic approaches.

Tripodo et al. [46] reported on a spatially resolved 53-gene signature comprising key genes of the dark-zone (DZ) mutational machinery, and light-zone (LZ) immune and mesenchymal milieu. This signature was applied to the transcriptomes of 543 cases of GCB-DLBCL and HGBCL-DH. According to the DZ/LZ signature, the GC-related lymphomas were sub-classified into two clusters. The subgroups differed in the distribution of DH cases and survival, with most DH cases displaying a distinct DZ-like profile. The clustering analysis was also performed using a 25-gene signature composed of DZ/LZ genes positively enriched in the non-B, stromal sub-compartments, for the first time achieving DZ/LZ discrimination based on stromal/immune features. The report offers new insight into the GC microenvironment, hinting at a DZ microenvironment of origin in DH lymphomas.

Intriguing research integrating transcriptomic, genetic, and immunophenotypic data of 347 DLBCLs demonstrated that MHC loss, particularly in GC-derived tumors originating from the centroblast-rich DZ, is associated with a strong enrichment of *EZH2* mutations, lower T cell infiltration, and poorer outcome [47]. Such results paved the way for the potential use of EZH2 inhibitors to treat the tumor by simultaneously modulating its immune microenvironment.

Finally, very recent work by Kotlov et al. [48] provided a relevant classification of DLBCL based on the transcriptomic characterization of TME from 4655 cases. Four major TME categories were identified as being associated with peculiar genetic/epigenetic aberrations of the malignant component, clinical behavior, and potential therapeutic targeting. Beyond its classification value, to date this work represents the most extensive translational and biological analysis of malignant and non-malignant DLBCL components.

By all means, in-depth TME analysis still represents an approach that can significantly improve the prognostication of DLBCL and even further tune the identification of different risk groups within the same COO category, predicting the response to targeted therapies.

## 4. Genetic Classification

Over the last few years, several proposals for a genetic classification of DLBCL have been published. Hereunder, the main contributions will be summarized and discussed based on the technical approach used.

### 4.1. Whole-Exome Sequencing (WES)-Based Studies

In 2017, Reddy and coworkers [49] reported on the whole-exome sequencing (WES) of 1001 FF DLBCLs and 400 paired germline DNAs. They found 150 driver genes to be recurrently mutated. The 60 top genes frequently exhibited a pattern of either predominant missense and/or copy number gains consistent with an oncogene or truncating mutations and/or copy number losses consistent with a tumor suppressor gene. When the mutational pattern was matched with the COO, 20 genes were differentially mutated between the two groups, including *EZH2, SGK1, GNA13, SOCS1, STAT6,* and *TNFRSF14*, which were mutated in GCB tumors, and *ETV6, MYD88, PIM1* and *TBL1XR1*, which were mutated in ABC tumors. Interestingly, *MLL2* mutations were associated with those of *MYC*, while *TP53* mutations occurred in a mutually exclusive fashion with *KLHL6*. CRISPR screening revealed that knockout of *EBF1*, *IRF4*, *CARD11*, *MYD88,* and *IKBKB* was selectively lethal in ABC DLBCL cell lines, as was knockout of *ZBTB7A, XPO1, TGFBR2*, and *PTPN6* in the GCB lines. On prognostic grounds, *MYC* mutations were strongly associated with poorer survival, as were mutations in *CD79B* and *ZFAT*. Mutations in *NF1* and *SGK1* were associated with more favorable survival. Furthermore, in ABC DLBCLs, genetic alterations in *KLHL14, BTG1, PAX5*, and *CDKN2A* were associated with significantly poorer survival, while those in *CREBBP* were associated with favorable outcomes. In the GCB-DLBCL group, genetic alterations in *NFKBIA* and *NCOR1* were associated with poorer prognosis, while alterations in *EZH2, MYD88*, and *ARID5B* were all associated with a significantly better prognosis. The authors developed a multivariate supervised learning approach for defining the association of survival with combinations of genetic markers (150 genetic driver genes) and gene expression markers (cell of origin, MYC, and BCL2). This led to the proposal of a three-subgroup molecular risk model that was found to outperform all existing predictors (i.e., COO, MYC/BCL2 DE, and IPI). However, the recent application of this model to 499 DLBCLs by Bolen et al. [50] did not provide independent validation. This might reflect the technical differences between the two studies (WES of FF samples by Reddy et al. vs. targeted NGS of DNA extracted from FFPE biopsies by Bolen et al.).

Two studies published in 2018 proposed a molecular subclassification of DLBCLs that had potential prognostic and therapeutic implications [51,52]. They both were based on WES and copy-number analysis of a large series of FF DLBCLs (304 and 574, respectively) [51,52].

Chapuy et al. [51] described five clusters characterized by different genetic lesions that were capable of identifying subgroups within the COO categories showing different behaviors.

Most cases included in clusters (Cs) 1 and 5 were classified as ABC. However, they showed important differences on molecular and prognostic grounds. C1 cases were thought to derive from marginal-zone B-cells, as they showed a stable mutational pattern, structural variants (SVs) of *BCL6*, and mutations of genes involved in the NOTCH2 and NF-kB pathways (*NOTCH2, SPEN, BCL10, TNFAIP3*, and *FAS*). Besides the multiple genetic lesions of genes involved in immune escape (*BM2, CD70, FAS, PD-L1, PD-L2*), these C1 cases carried *MYD88* mutations which were non-L265P, unlike what was observed in the cases included in C5. Notably, C1 cases had a rather favorable course and revealed potential therapeutic targets related to NOTCH2 and BCL6 signaling and immune evasion mechanisms. C5 tumors, which behaved more aggressively than the C1 ones, showed mutations of *MYD88^L265P^*, *CD79B, PIM1, TBL1XR1, GRHPR,* and *BTG1*, SV of 18q, and activation of the NF-kB pathway. In addition, they carried ongoing mutations, being at least in part under the effect of AID. Potential targets for C5 cases corresponded to BCR/TLR signaling and BCL2.

Cs 3 and 4 were significantly enriched in GCB cases but were characterized by different genetic lesions and responses to chemoimmunotherapy. The majority of DLBCLs in C3 harbored *BCL2* mutations with concordant SVs. They also exhibited frequent mutations in chromatin modifiers, *KMT2D*, *CREBBP*, and *EZH2*, and increased transcriptional abundance of EZH2 targets by gene set enrichment analisys (GSEA). These tumors also had alterations in the B-cell transcription factors *MEF2B* and *IRF8*, and indirect modifiers of BCR and PI3K signaling (*TNFSF14*(*HVEM*), *HCNV1*, and *GNA13*). In addition, C3 tumors had two alternative mechanisms of inactivating *PTEN*: focal *10q23.31/PTEN* loss and predominantly truncating *PTEN* mutations, events that play a role in the process of lymphomagenesis. C4 DLBCLs were characterized by mutations in four linker and four core histone genes, multiple immune evasion molecules (*CD83*, *CD58*, and *CD70*), BCR/PI3K signaling intermediates (*RHOA*, *GNA13*, and *SGK1*), NF-kB modifiers (*CARD11*, *NFKBIE*, and *NFKBIA*), and RAS/JAK/STAT pathway members (*BRAF* and *STAT3*). Comparison of the C3 and C4 genetic signatures further revealed that these GCB-DLBCLs utilized distinct mechanisms to perturb common pathways such as PI3K signaling. In contrast to C3 DLBCLs, C4 tumors rarely exhibited *PTEN* alterations but harbored more frequent *RHOA* mutations. In addition, C4 DLBCLs rarely exhibited *BCL2* alterations and had higher mutational density. The distinct genetic features of C3 and C4 GCB-DLBCLs led Chapuy et al. to suggest specific targeted therapies including inhibition of BCL2, PI3K, and the epigenetic modifiers EZH2 and CREBBP in C3 GCB tumors, and JAK/STAT and BRAF/MEK1 blockade in C4 GCB-DLBCLs. Last but not least, C3 cases had a far worse prognosis.

C2 DLBCLs harbored frequent biallelic inactivation of *TP53* by mutations and *17p* copy loss. In addition, they often exhibited copy loss of *9p21.13/CDKN2A* and *13q14.2/RB1*, perturbing chromosomal stability and cell cycle. C2 tumors also had significantly more driver somatic copy number alterations (SCNAs) and a higher proportion of genome doubling events. This cluster included both GCB- and ABC-DLBCLs, as did prior DLBCL cohorts with *TP53* mutations in targeted analyses [53]. Prognostically significant SCNAs, including *13q31.31/miR-17-92* copy gain and *1q42.12* copy loss, were also more common in these DLBCLs, which were characterized by a rather unfavorable prognosis.

A further cluster, termed 0, was also detected, which apparently lacked significant genetic alterations. However, as the C0 group consisted almost exclusively of T-cell rich/histiocyte-rich B-cell lymphomas, the obtained results might have been largely influenced by the small number of neoplastic cells.

The authors further evaluated *BCL2* and *MYC* alterations. Tumors with cooccurring *BCL2* and *MYC* SVs were significantly more frequent in C3 DLBCLs.

Importantly, the coordinate genetic signatures reported by Chapuy et al. predicted outcomes independent of IPI which could suggest new combination treatment strategies and, more broadly, provide a roadmap for actionable DLBCL classification [51].

By their integrated approach, Schmitz et al. [52] identified four prominent genetic subtypes among 574 DLBCLs which they termed MCD (based on the co-occurrence of *MYD88^L265P^* and *CD79B* mutations), BN2 (based on *BCL6* fusions and *NOTCH2* mutations), N1 (based on *NOTCH1* mutations), and EZB (based on *EZH2* mutations and *BCL2* translocations). Interestingly, Schmitz and co-workers enriched their series with unclassified DLBCLs. The latter turned out to frequently carry mutations affecting *SPEN* and *NOTH2* as well as *BCL6* fusions. ABC cases were enriched in *MYD88^L265P^* and *CD79B* or *NOTCH1* mutations, with the two conditions being mutually exclusive. GCB tumors showed the co-occurrence of *EZH2* mutations and *BCL2* translocations. The MCD and N1 subtypes were dominated by ABC cases, while EZB included mostly GCB tumors, and BN2 had contributions from all GEP subgroups. Overall, about 45% of the samples were classified into the genetically pure subtypes of DLBCL.

The MCD subtype displayed 82% of cases carrying *MYD88*^L265P^ or *CD79B* aberrations (mutation or amplification), with 42% bearing both abnormalities. The MCD subtype showed a frequent gain or amplification of *SPIB*, encoding a transcription factor that, with IRF4, defines the ABC phenotype and promotes plasmacytic differentiation. Known tumor suppressors in MCD include *CDKN2A*, *ETV6*, *BTG1*, and *BTG2*, and putative tumor suppressors include *TOX*, *SETD1B*, *FOXC1*, *TBL1XR1*, and *KLHL14*. The tumor suppressor *TP53* was mutated significantly less often in MCD as compared to other subtypes. Immune editing appeared prominent in MCD genomes, with 76% acquiring a mutation or deletion of *HLA-A*, *HLA-B*, or *HLA-C* and 30% acquiring truncating mutations targeting CD58.

BN2 was dominated by NOTCH pathway aberrations, with 73% acquiring a *NOTCH2* mutation or amplification, *SPEN* mutation, or mutation in *DTX1*, a NOTCH target gene. *BCL6* fusion, the other BN2 hallmark, occurred in 73% of cases. *BCL6* fusions were enriched in cases with *NOTCH2*, *SPEN*, or *DTX1* lesions to a significantly greater extent in BN2 than in non-BN2 cases. Genetic aberrations (mutations or amplifications) targeting regulators of the NF-kB pathway were a prominent feature of BN2. These more often affected *TNFAIP3*, *PRKCB*, and *BCL10*. Other likely gain-of-function events included mutations targeting *cyclin D3* and *CXCR5*, whereas inactivating lesions targeting the immune regulator *CD70* suggested immune escape.

N1 was characterized by *NOTCH1* mutations and aberrations targeting transcriptional regulators of B-cell differentiation (*IRF4*, *ID3*, and *BCOR*), which may contribute to its plasmacytic phenotype. *TNFAIP3* mutations in N1 could reinforce this phenotype by fostering NF-KB-induced IRF4 expression.

EZB was enriched for most of the genetic events previously ascribed to GCB-DLBCL, including *BCL2* translocation, *EZH2* mutation, and *REL* amplification, as well as inactivation of the tumor suppressors *TNFRSF14*, *CREBBP*, *EP300*, and *KMT2D*. The germinal-center homing pathway involving *S1PR2* and *GNA1314* was disrupted in 38% of EZB cases. JAK-STAT signaling was promoted in about half cases by a *STAT6* mutation or amplification or by a mutation or deletion targeting *SOCS1*. PI3K target of rapamycin signaling turned out to be activated in 23% of cases by *MTOR* mutations or the amplification of *MIR17HG*. Immune editing was of interest in EZB genomes since 39% acquired lesions in the major histocompatibility complex class II pathway genes *CIITA* and *HLA-DMA*.

The four subtypes differed significantly in PFS and OS, with the BN2 and EZB subtypes having much more favorable outcomes than the MCD and N1 subtypes. The predicted 5-year OS rates for the MCD, N1, BN2, and EZB subtypes were 26%, 36%, 65%, and 68%, respectively. Within ABC DLBCL, patients with MCD had significantly inferior survival as compared with those with BN2, and patients with either MCD or N1 had significantly inferior survival as compared with patients with ABC tumors that were not genetically classified. Within GCB-DLBCL, there was a trend toward inferior OS among patients with EZB as compared with those with other GCB tumors. The COO subgroups and genetic subtypes independently contributed to survival in a multivariate analysis. Conversely, the IPI score did not vary significantly among the genetic subtypes, but the latter significantly added to IPI. A trend toward increased extranodal involvement (e.g., CNS) was a feature of MCD, which reflected the frequent *CD79B* and *MYD88*^L265P^ mutations.

On therapeutic grounds, constitutive BCR signaling activation was most frequent in MCD and least frequent in EZB, but genetic alterations involving the BCR cascade occurred in all genetics subtypes, suggesting that constitutive BCR signaling is a pervasive aspect of DLBCL pathogenesis. BN2 was notably enriched for BCR–NF-kB and IKK regulator aberrations. In addition to NF-kB, survival of DLBCL cells turned out to be promoted by antiapoptotic BCL2 family members, which were targeted by genomic amplification or translocation in 17.4% of cases. As expected, BCL2 mRNA levels were significantly higher in EZB tumors with *BCL2* translocations than in other EZB tumors. MCD tumors also had high BCL2 mRNA expression as compared with other cases, a finding due to mechanisms other than translocation or amplification.

### 4.2. Targeted NGS and Bioinformatic-Based Studies

Lacy et al. [54] applied a 293-gene chip to DNA extracted from FFPE tissue samples by using a Covaris LE220. The authors sequenced a large, unselected cohort consisting of 928 DLBCL patients all treated with R-CHOP and provided with full clinical follow-up. Bernoulli mixture-model clustering was applied, and the resulting subtypes analyzed in relation to their clinical characteristics and outcomes. Five molecular subtypes were resolved, termed MYD88, BCL2, SOCS1/SGK1, TET2/SGK1, and NOTCH2, along with an unclassified group. The subtypes characterized by genetic alterations of *BCL2*, *NOTCH2*, and *MYD88* recapitulated the above-mentioned studies showing good, intermediate, and poor prognosis, respectively. The SOCS1/SGK1 subtype showed biological overlap with primary mediastinal B-cell lymphoma and conferred excellent prognosis. Although not identified as a distinct cluster, *NOTCH1* mutation was associated with poor prognosis. The impact of *TP53* mutation varied with genomic subtypes, conferring no effect in the NOTCH2 subtype and poor prognosis in the MYD88 subtype. The results obtained by Lacy et al. are summarized in Table 2, where they are also compared with the subtypes reported by Chapuy et al. [51], and Schmitz et al. [52].

Ennishi et al. [55] performed an integrative genomic and transcriptomic analysis of DLBCL using a British Columbia population-based registry. They uncovered recurrent biallelic *TMEM30A* loss-of-function mutations which were associated with a favorable outcome and were uniquely observed in DLBCL. Using *TMEM30A-*knockout systems, increased accumulation of chemotherapy drugs was observed in *TMEM30A-*knockout cell lines and *TMEM30A-*mutated primary cells, accounting for the improved treatment outcome. Furthermore, they found increased tumor-associated macrophages and an enhanced effect of anti-CD47 blockade limiting tumor growth in *TMEM30A-*knockout models. By contrast, Ennishi et al. showed that TMEM30A loss-of-function increased B-cell signaling following antigen stimulation—a mechanism conferring selective advantage during B-cell lymphoma development. These findings suggested intrinsic and extrinsic vulnerabilities of cancer cells that can be therapeutically exploited.

Finally, Wright et al. [56] developed an algorithm to determine the probability of a patient’s lymphoma belonging to one of seven genetic subtypes based on its genetic features. This represented a probabilistic classification tool (LymphGen) using any combination of mutational, copy number, and *BCL2/BCL6* rearrangement data. Schmitz’s cohort was used as training set, while those from Chapuy et al. and Ennishi et al. were used for validation. Wright et al. developed a model that is summarized in Table 3, which also includes information on potential therapeutic targets related to the genetic subtype of DLBCL.

## 5. Open Issues and Perspectives

The paper of Lacy et al. [54] was accompanied by a commentary from Morin and Scott [57], who concluded that comprehensive sequencing of a larger number of tumors with the combination of whole-genome and transcriptome sequencing is warranted to develop a new molecular taxonomy which may be concretely translated into clinical benefits. In fact, between 7.5% and 55% of the cases reported by Chapuy et al., Schmitz et al., and Lacy et al. did not fit into any of the major genetic categories they identified [51,53,58]. The fact that the genomic studies hitherto reported show a certain variability in terms of results may depend on different factors, such as the size of the analyzed cohort or heterogeneity of the techniques used (e.g., FF vs. FFPE tissue, whole exome vs. targeted sequencing, and the statistical approach applied), but also on the actual heterogeneity of the lesions occurring in these tumors. For instance, divergent evolution within the same biopsy, which corresponded to different morphologic, phenotypic, and COO features [59], has been reported. Although the distinct components had a common clonal origin and shared the bulk of genetic aberrations, each revealed private mutations, in keeping with the above-mentioned morpho-phenotypic and molecular differences.

The heterogeneity of genetic lesions is much greater than was thought until a couple of years ago. This has been highlighted by liquid biopsy (LB) [59,60,61]. By ultradeep sequencing of the cell-free circulating tumoral DNA (cfDNA) released by neoplastic cells undergoing apoptosis, it has been shown that the global mutational landscape of DLBCL is indeed wider than that observed in diagnostic biopsies, which means that different mutations can occur at different anatomic sites. Once a standardized methodology is developed and the cost per test is reduced, LB can represent a real-time noninvasive tool for disease monitoring. In fact, patients achieving early molecular response (a 2-log decrease of ctDNA after one cycle of standard chemoimmunotherapy) and major molecular response (a 2.5-log decrease after two cycles) show a significantly superior outcome at 24 months independently of IPI and interim positron emission tomography. Conversely, among treatment-resistant subjects, new mutations are acquired in cfDNA, marking resistant clones selected during the clonal evolution.

The continuous development of sequencing and bioinformatic techniques will allow us to achieve the long searched-for goal of using customized therapies based on the molecular characteristics of each individual tumor. Some approaches do appear to be more easily and cheaply applicable to daily life. Nevertheless, the more comprehensive the bioinformatic approach is, the higher the likelihood of overcoming today’s standard chemoimmunotherapy and designing chemotherapy-free protocols capable of curing most (if not all) patients, with minimal or no toxic effects.

## Figures and Tables

**Table 1:** Diffuse large B-cell lymphoma, high-grade B-cell lymphoma, and gray-zone lymphoma according to the Revised Fourth Edition of the WHO Classification of Tumours of Haematopoietic and Lymphoid Tissues (italics indicate that an entity is provisional).

**Diffuse Large B-Cell Lymphoma (DLBCL):**
DLBCL not otherwise specified (NOS)
Morphological variants
Centroblastic
Immunoblastic
Anaplastic
Other rare variants
Molecular subtypes
Germinal centre B-cell subtype (GCB)
Activated B-cell subtype (ABC)
**Other lymphomas of Large B-Cells:**
T-cell/histiocyte-rich large B-cell lymphoma
Primary DLBCL of the CNS
Primary cutaneous DLBCL, leg type
EBV-positive DLBCL, NOS
EBV-positive mucocutaneous ulcer
DLBCL associated with chronic inflammation
Lymphomatoid granulomatosis
Large B-cell lymphoma with *IRF4* rearrangement
Primary mediastinal (thymic) large B-cell lymphoma
Intravascular large B-cell lymphoma
ALK-positive large B-cell lymphoma
Plasmablastic lymphoma
*HHV8-positive DLBCL*
Primary effusion lymphoma
**High-Grade B-Cell Lymphoma:**
High-grade B-cell lymphoma with *MYC* and *BCL2* and/or *BCL6* rearrangement
High-grade B-cell lymphoma, not otherwise specified (NOS)
**B-cell lymphoma, unclassifiable, with features intermediate between DLBCL and classic Hodgkin’s lymphoma**

**Table 2:** Molecular subtypes of DLBCL according to Lacy et al. in comparison with those of Chapuy et al. and Schmitz et al.

Lacy et al.		Chapuy et al.		Schmitz et al.			Notes
MYD88	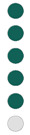	C5	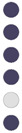	MCD	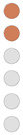	*MYD88*	Strongly associated with ABC. The most robust group in all reports. Contains the most primary PCNSL and testicular lymphoma. Poor prognosis.
						*CD79B*	
						*PIM1*	
						*ETV6*	
						*CDKN2A*	
						*TBL1XR1*	
BCL2	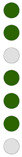	C3	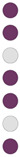	EZB	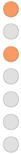	*EZH2*	Strongly associated with GCB. Contains most transformed FLs and cases with a concurrent FL. Generally favorable prognosis, although enriched for cases of double-hit lymphoma and MHG.
						*BCL2*	
						*BCL2 translocation*	
						*KMT2D*	
						*TNFRSF14*	
						*CREBBP*	
						*CREBBP2*	
SOCS1/SGK1	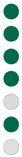	C4	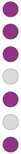		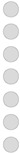	*CD83*	Predominantly GCB. Shares genetic and gene expression features of PMBCL. Associated with the most favorable prognosis.
						*HIST1H1E*	
						*SGK1*	
						*NFKBIA*	
						*NFKBIE*	
						*SOCS1*	
						*BRAF*	
TET2/SGK1	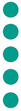		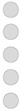		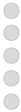	*TET2*	A less strongly identifiable subtype. Has very strong similarity to SOCS1/SGK1 but differs by the addition of TET2 and BRAF and the lack of SOCS1 and CD83. Favorable prognosis.
						*BRAF*	
						*SGK1*	
						*KLHL6*	
						*ID3*	
NOTCH2	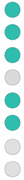	C1	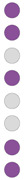	BN2	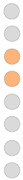	*BCL10*	Not associated with any COO. Shares mutational similarity to MZL but not enriched for transformed MZLs. Less strongly defined than other subgroups.
						*TNFAIP3*	
						*NOTCH2*	
						*BCL6 translocation*	
						*CCND3*	
						*SPEN*	
						*UBE2A*	
						*CD70*	
NEC	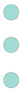		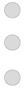	Other	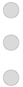	*NOTCH1*	A default category, containing cases that could not be classified elsewhere and no detected mutation. Likely to also contain cases belonging to both NOTCH1 and TP53/CNA subgroups.
						*REL amplification*	
						*TP53*	
	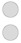	C2	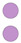		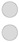	*TP53*	Characterized by TP53 mutation and widespread copy number changes.
						*Frequent deletions*	
	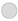	C0	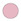		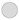	*No detected abnormalities*	Cases with no detectable mutation were allocated to the NEC group.
	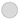		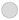	N1	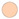	*NOTCH1*	Characterized by NOTCH1 mutation, this was significantly elevated in Lacy’s NEC group but only mutated in 2.5% of samples. Associated with poor outcome.

PCNSL, primary central nervous system lymphoma; FL, follicular lymphoma; MHG, molecular high grade; MZL, marginal zone lymphoma.

**Table 3:** Implications of genetic subtypes of DLBCL for therapy (from Wright et al., modified).

		*Genetics*	*GEP Signature*	*Related LNH*	*Targets*	*5y-OS*
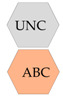	*MCD*	My-T-BCR dependent NF-kB; Immune evasion-MHC class I; Cell survival; BCL2 expression; Altered B-cell differentiation; G1-S cell cycle/p53 checkpoint; BCR: IgM >> IgG; Ig_VH_ 4-34^++^	B-cell activation NF-kB IRF4 MYC Proliferation	Primary extranodal DLBCL Transformed WM	BCR-dep. NF-kB PI3K mTORC1 BCL2-BCLX_L-_MCL1 JAK1 IRAK4 IRF4	40% All 37% ABC
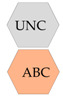	*N1*	NOTCH1 signaling Altered B cell differentiation BCR: IgM > IgG	NOTCH Quiescence Plasma cells T cell-Myeloid-FDC	NOTCH1 mutated CLL	NOTCH1 Immune checkpoints	27% All 22% ABC
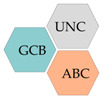	*A53*	TP53 inactivation—DNA Damage Aneuploidy Immune evasion—*B2M* loss BCR: IgM >> IgG; Ig_VH_ 4-34^++^	p53 Immune low	-	BCR-dep. NF-kB	63% All 33% ABC 100% GCB
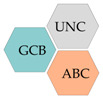	*BN2*	NOTCH2 signaling Altered B cell differentiation BCR-dependent NF-kB Immune evasion—*CD70* loss Proliferation—Cyclin D3 BCR: IgM >> IgG; Ig_VH_ 4-34^++^	B-cell activation NF-kB NOTCH Proliferation	MZL Transformed MZL	BCR-dep. NF-kB PI3K mTORC1 BCL2 NOTCH2	67% All 76% ABC 100% GCB 38% UC
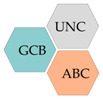	*ST2*	JAK/STAT3 signaling NF-kB activation *P2RY8-GNA13* activation Altered B cell differentiation BCR: IgM >> IgG	GC B cell PI3K signaling JAK2 signaling Glycolysis Stromal	NLPHL THRLBCL	PI3K JAK2	84% All 81% GCB
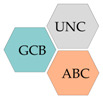	*MYC^+^*	Chromatin modification Anti-apoptosis PI3K signaling *S1PR2-GNA13* inactivation Altered T_FH_ interactions MYC (EZ-MYC^+^) BCR: IgG > IgM	GC LZ (MYC^−^) GC IZ (MYC^+^) BCL6 (MYC^+^) TCF3 (both) T_FH_ cells (MYC^−^) Stromal (MYC^−^) Immune low (MYC^+^)	FL Transformed FL BL (EZB-MYC^+^)	PI3K mTORC1 EZH2 BCL2-MCL1	48% MYC^+^ 82% MYC^−^
	*MYC^−^*					

WM, Waldenström macroglobulinaemia; CLL, chronic lymphocytic leukemia; MZL, marginal zone lymphoma; NLPHL, nodular lymphocyte-predominant Hodgkin lymphoma; THRLBCL, T cell histiocyte-.
